# Evaluation of mechanical accuracy for couch‐based tracking system (CBTS)

**DOI:** 10.1120/jacmp.v13i6.3818

**Published:** 2012-11-08

**Authors:** Suk Lee, Kyung‐Hwan Chang, Jand Bo Shim, Yuanjie Cao, Chang Ki Lee, Sam Ju Cho, Dae Sik Yang, Young Je Park, Won Seob Yoon, Chul Yong Kim

**Affiliations:** ^1^ Department of Radiation Oncology College of Medicine Korea University Seoul Korea; ^2^ Department of Biomedical Engineering College of Medicine Korea University Seoul Korea; ^3^ Department of Information and Communication ShinGyeong University Gyeonggi‐Do Korea; ^4^ Department of Radiation Oncology College of Medicine Eulji University Seongnam Korea

**Keywords:** couch, tracking, image‐guided radiation therapy (IGRT), delay time, consistency

## Abstract

This study evaluated the mechanical accuracy of an in‐house–developed couch‐based tracking system (CBTS) according to respiration data. The overall delay time of the CBTS was calculated, and the accuracy, reproducibility, and loading effect of the CBTS were evaluated according to the sinusoidal waveform and various respiratory motion data of real patients with and without a volunteer weighing 75 kg. The root mean square (rms) error of the accuracy, the reproducibility, and the sagging measurements were calculated for the three axes (X, Y, and Z directions) of the CBTS. The overall delay time of the CBTS was 0.251 sec. The accuracy and reproducibility in the Z direction in real patient data were poor, as indicated by high rms errors. The results of the loading effect were within 1.0 mm in all directions. This novel CBTS has the potential for clinical application for tumor tracking in radiation therapy.

PACS number: 87.55.ne

## I. INTRODUCTION

Image‐guided radiation therapy (IGRT) techniques to compensate for tumor motion can improve local control by increasing the dose escalation while minimizing the internal target volume (ITV) margin and normal tissue complication probability.^(1)^

Intrafraction motions due to respiration in the thoracic and abdominal regions have been observed.^1^, ^3^ Respiration‐induced tumor motion can result in radiation‐induced toxicity in healthy tissue.^(2)^ The American Association of Physicists in Medicine Task Group 76^(3)^ recommends the use of appropriate respiratory motion management methods if the target motion is greater than 5 mm in any direction during beam delivery.

IGRT with motion management is a technique that synchronizes radiation delivery with respiration by movement of a linear accelerator or the patient such that the radiation beam follows the tumor during treatment.^(3)^ Motion management requires both acquisition and correction techniques. Acquisition techniques incorporate the following tools: methods that acquire signals and images using a spirometer combined with artificial manipulation of the patient's respiration;^4^, ^8^ sensors that are used in real‐time position management (RPM), such as the Anzai, Calypso, and ExacTrac systems; fluoroscopy; and cone‐beam computed tomography (CBCT).^9^, ^17^

Several methods, including acquisition and correction techniques, can be used to manage respiration‐induced tumor motion. Acquisition techniques can be classified into three groups: respiration‐based, sensor‐based, and image‐based techniques. Hanley et al.^(4)^ reported a PTV margin decrease from 1–2 cm to 0.2–0.5 cm using deep inspiration breath‐holding (DIBH) for lung tumors. The authors demonstrated that the target dose escalation improved because of the decreased response of normal lungs to high doses. Wong et al.^(7)^ demonstrated that the excursion of the target center between two patients at the same ABC phase was less than 1 mm for liver cases. Christensen et al.^(8)^ reported that image registration and spirometry can reduce the radiation dose to normal tissue by minimizing the tumor treatment margins, although a model for predicting tumor motion should first be developed. Wagman et al.^(9)^ confirmed that the calculated dose of approximately 21% increased because the margin reduction in the gross tumor volume (GTV) to PTV decreased from 2 cm to 1 cm when using the RPM. Li et al.^(10)^ demonstrated that the Anzai gating system is robust and can compensate for respiration motion during radiation therapy. The authors suggested that the success of the treatment using this gating system depended on patients because the treatment time was two‐to‐three times longer than the treatment time required for conventional radiation therapy. Another study^(11)^ compared the tumor volume and tumor position using the RPM system and AZ‐733V systems, and documented a difference in tumor volume between the two systems exceeding 25%. It was also determined that the tumor position did not differ significantly. Willoughby et al.^(12)^ reported that the accuracy of the Calypso system for localization was 1.5±0.9  mm compared with two different KV X‐ray localization methods for prostate motion. A study of the localization accuracy of the ExacTrac X‐ray 6D system revealed that the localization accuracy of the 6D fusion algorithm was less than 1 mm and 2.5°.^(13)^ These results suggested that using the ExacTrac X‐ray 6D system can increase the localization accuracy for patients and may reduce the internal margin. Another study^(14)^ investigated the difference in the marker position between manual setup using skin markers and setup using a fluoroscopic X‐ray system for the prostate. It reported that dislocation was decreased by less than 1 mm for the lateral, anteroposterior, and craniocaudal directions using the RTRT system. The authors suggested that an excessive exposure dose from the fluoroscopic X‐ray system should be prevented and also indicated the need for further studies to determine the marker position that minimizes the PTV margins and enables refinement in the design of PTV margins. Another study found the margins to completely encompass tumors ranging in diameter from 0.7 to 1.7 cm using four‐dimensional computed tomography (4DCT).^(15)^ Hammoud et al.^(16)^ reported that the difference between the maximal border displacement of the planning CT and CBCT ranged from 79% to 99%, within 5 mm in all directions in prostate localization. An investigation of the accuracy and precision of four‐dimensional ultrasound (4DUS) to track respiration‐induced liver motion confirmed that the 4DUS‐based tracking system has the potential to be used with motion management techniques to manage respiration‐induced motion.^(17)^

Correction techniques can be categorized into three groups: gating for the machine, real‐time tracking, and gating for the patient (breath‐holding). The gating technique continuously monitors the respiration cycle of the patient and the tumor motion due to the patient's breathing during radiation therapy and compensates by turning the beam on and off. The respiratory gating technique has several disadvantages, including an increased treatment time caused by the intermittent beam application.^(9)^ The radiation dose to patients may be increased due to the additional imaging dose, and pulmonary complications, such as pneumothorax, may occur because of the insertion of the fiducial marker.

The real‐time tracking system repositions the radiation beam to follow the changing position of the tumor via a multileaf collimator (MLC)^18^, ^21^ and couch movement^(22)^. An MLC‐based tracking system can reduce the treatment time compared with gating techniques and can improve patient safety and comfort.^(20)^ Correction techniques can be classified into three groups: gating for the machine, real‐time tracking, and gating for the patient (breath‐holding). In the beam on/ off methods, the beam is only turned on within a predefined window of the patient's respiration cycle. Tracking methods can be classified as MLC‐based and couch‐based tracking methods. In one study, the reduction of the PTV margin using MLC tracking was 2.72±0.43  mm (cranial side) and 1.66±0.13  mm (caudal side) for a 15 mm peak‐to‐peak displacement, and 5.46±1.15  mm (cranial side) and 3.58±1.15  mm (caudal side) for a 25 mm peak‐to‐peak displacement.^(18)^ Another study reported good geometric accuracy and higher dosimetric conformality for real‐time 3D dynamic multileaf collimator (DMLC)‐based tumor tracking.^(19)^ The authors found that the DMLC tracking system compensated for the intrafraction motion, and suggested that a faster MLC is needed to increase the efficiency of beam delivery. Finally, another study demonstrated that tumor motion can be compensated for by using couch‐based tumor tracking techniques for step‐and‐shoot delivery, fixed fields, and dynamic arc delivery cases.^(20)^

The aim of this study was to evaluate the mechanical accuracy of the couch‐based tracking system (CBTS) by measuring the accuracy, consistency, delay time, and sagging of the CBTS for clinical applications.

## II. MATERIALS AND METHODS

**Figure 1:**
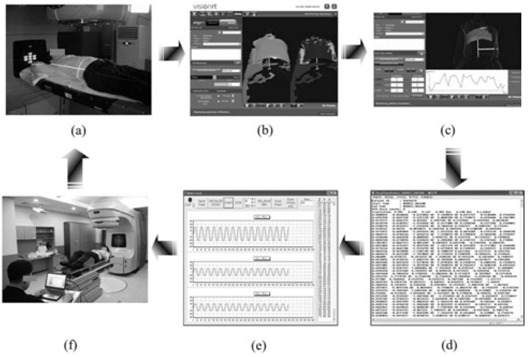
The tracking procedure of the couch‐based tracking system (CBTS). A volunteer was positioned on the treatment table of a linear accelerator (a). After the surface position information of the patient using AlignRT was acquired (b, c), the data were transferred to the tracking program of the CBTS (d, e). The CBTS is tracked according to the respiratory motion (f).

The CBTS process is presented in Figure 1. A volunteer was positioned on the treatment table of a linear accelerator (CL iX, Varian Medical Systems, Palo Alto, CA). The three‐dimensional (3D) surface data of the patient were acquired using AlignRT (version 4.0, Vision RT, London, UK), which is a video‐based 3D surface imaging system. The system consists of two 3D cameras mounted on the ceiling of the treatment room. After the surface position information of the patient was acquired, the data were transferred to the CBTS tracking program, after which the CBTS was tracked according to the respiratory motion.

### A. respiration data

#### A.1 Sinusoidal waveform

The sinusoidal waveform is a sine wave representing an artificial wave with a constant cycle. The sinusoidal waveform was recorded at a sampling period of 10 ms for 60 s. We evaluated the mechanical movement of the CBTS according to the sinusoidal waveform.

#### A.2 Various patient respiratory motion data

Various respiratory motion data were obtained using the AlignRT system. Real patient data included inhalation, exhalation, cough, and breath‐holding data. The respiration data were recorded at a sampling period of 10 ms for 60 s. The mechanical movement of the CBTS was evaluated according to the various respiration data.

### B. CBTS

Specifications of the in‐house–developed CBTS, the tracking program, and each component are shown in Table 1.

**Table 1:** Specifications for the couch‐based tracking system.

*Couch*	*Specifications*
Geometrical dimension	Couch dimension: 1700 mm × 472 mm × 211 mm
	Maximum amplitude: X direction (lateral): ±1.5 cm,
Y direction (longitudinal): ±1.5 cm, Z direction (vertical): ±2.0 cm
	Maximum duration weight: 100 kg
	Closed‐loop system or feedback control system
Control	Couch position acquisition: linear strip, resolution of 0.05 mm
	Communication system: RS‐232C^a^
	Communication speed: 115.2 kbps
	Resolution of linear encoder: ±0.127 mm
	Couch position control: PWM^b^ output in MCU^c^ module
	Analog‐to‐digital conversion time of MCU: 13–260 μs
	Control method: PID^d^ control algorithm
	Maximum velocity: 20 mm/s
	Formula of couch position control:
	Out(k)=Kp ^e^ x Err(k) + Ki^f^ x (Err(k)+Err(k‐1))+Kd^g^ x (Err(k^h^)‐Err(k‐1^h^))
Driving	Three BLDC^i^ motor and three drivers
Analysis	Sampling time: 10 ms
	5‐point simple moving average method

^a^ RS‐232=recommended standard 232 communication

^b^ PWM=pulse width modulation

^c^ MCU=microcontroller unit

^d^ PID=proportional‐integral‐derivative

^e^ Kp=proportional gain

^f^ Ki=integral gain

^g^ Kd=derivative gain

^h^k, k‐1=control step

^i^ BLDC=brushless direct current.

#### B.1 CBTS control component

**Figure 2:**
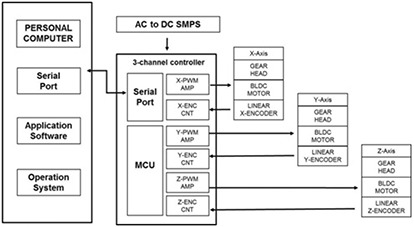
The circuit diagram of the controller component.

The circuit diagram of the control component consists of a microcontroller unit (MCU, ATmega8A, San Jose, CA), pulse width modulation (PWM), and a linear encoder (EM1 Transmissive optical encoder module, EM1‐0‐127, US DIGITAL, Vancouver, WA, resolution of ± 0.127 mm) (Fig. 2). The position data of the three axes were obtained using AlignRT. The surface position data of the patient were transferred to the serial port in the MCU through the RS‐232C communication system with a communication velocity of 115.2 kbps at an interval of 0.01 s. The MCU generated an optimized control signal and produced the motor control signals in real time using a PID algorithm capable of manipulating the process inputs based on the history and the rate of change of the signal according to the following equation:

(1) out(k)=Kp×Err(k)+Ki×(Err(k)+Err(k−1))+Kd×(Err(k)−Err(k−1))

where *Out (k)* is the current position as controller output, *k* is the present position, *Kp* is the proportional gain, *Err* is the error, *Ki* is the integral gain, and *Kd* is the derivative gain.

OS=operation system; AC=alternating current; DC=direct current; PWM=pulse width modulation; ENC=encoder; CNT=controller; MCU=microcontroller unit

#### B.2 CBTS driving component

**Figure 3:**
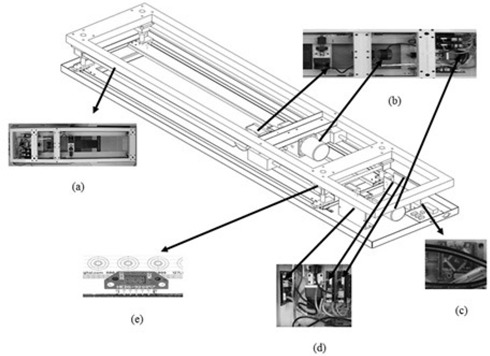
The driving component of the couch‐based tracking system: (a) the structure of the couch‐based tracking system; (b) three BLDC motors; (c) MCU (micro controller unit); (d) three BLDC motor drivers; (e) transmissive linear strip and transmissive optical encoder.

The driving component consists of three brushless direct current (BLDC) motors (BD‐0‐N2202200; SEWOO Industrial Systems, Seoul, Korea) and three BLDC drivers (SBDSMS‐03A; SEWOO Industrial Systems) (Fig. 3). The control signal produced in the MCU is transferred to the three motors. The CBTS tracks the surface position data of the three directions using three motors. The current couch position information in relation to the three axes was fed back to the tracking program using the linear encoder and a transmissive linear strip (LIN‐120‐0.5‐N; US DIGITAL, Vancouver, WA) at a resolution of 0.05 mm.

#### B.3 CBTS tracking program

Data display and analysis were performed using a tracking program written in Delphi (Inprise, Kansas City, MO). First, we verified the interface between the tracking program and the CBTS. By clicking on the initialize couch button, the couch is positioned at the default position (i.e., X=0, Y=0, and Z=0). Three types of respiration data are then imported. The sine‐wave gen button imports the sinusoidal waveform, the Excel direct button imports the real patient data, and the Excel 5‐point avg button produces the average of the real patient data, using a 5‐point simple moving averaging (SMA) method that calculates an average of the actual value of the previous five periods as follows:

(2) $$M_{t}=\frac{1}{N}(X_{t}+X_{t-1}+X_{t-1}+\cdot \cdot +X_{t-N+1})$$

where $M_{t}$ represents the simple average value from t‐N+1 to t, *N* represents the total number of periods in the average, and *X* represents the actual value of the current point. The acquisition data show the respiration pattern of the patient converted into a waveform and the tracking data (blue line) of the CBTS with respect to the respiration data. Both the respiration data and the tracking data are shown in the lateral (X), the longitudinal (Y), and the vertical (Z) directions. The stop button ends the couch tracking, after which time the couch is repositioned to the default setting.

### C. CBTS Evaluation

#### C.1 Delay time

The CBTS has a mechanical delay time between the respiration data and the tracking data and an inherent processing time during which the respiration data are transferred to the serial port within the MCU through the RS‐232C communication system with a communication velocity of 115.2 kbps. To quantitatively analyze the respiration data and tracking data with the waveform displayed on the tracking program screen, we changed two datasets to the text file format. We then calculated the average delay time from the phase (time) difference between two curves using an experiment that measures the accuracy, reproducibility, and loading effect of the CBTS. A respiration dataset in three directions (X, Y, and Z) was calculated as 12‐byte data when the MCU received the respiration data. The processing time was calculated as 120‐bit data in our system because the RS‐232C communication system uses asynchronous transmission in which the transmitted data are encoded with start and stop bits, specifying the beginning and end of each character. Therefore, we can calculate the processing time using the RS‐232C data transfer rate. The overall delay time of the CBTS was calculated as the sum of the two delay times.

#### C.2 Accuracy

**Figure 4:**
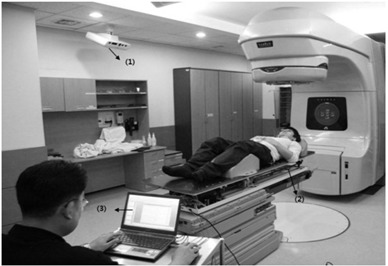
The couch‐based tracking system (CBTS) after patient set‐up: (1) the 3D camera of the AlignRT system; (2) the CBTS; (3) the tracking program of the CBTS.

As shown in Fig. 4, the couch was positioned on the treatment table of the medical linear accelerator. To measure the accuracy of the CBTS according to the two respiration data sets (sinusoidal waveform and various respiratory motion data), we used the CBTS and the tracking program and performed the measurements by monitoring the respiration data and tracking data with and without a volunteer weighing 75 kg (Fig. 4). The measurement was repeated at intervals of 60 s for 3 min. After the two datasets were converted into text files, we calculated the root mean square (rms) error of the accuracy in three directions based on the phase difference between the respiration data and the tracking data.

#### C.3 Reproducibility

The consistency measurement of the CBTS was performed using the same setup as the reproducibility measurement. In the case of the sinusoidal waveform, we analyzed the reproducibility for 60 s cycles. In the case of real patient data, we analyzed the cycle reproducibility, including the inhalation, exhalation, cough, and breath‐holding data. After the data were converted into text files, we calculated the rms error of the reproducibility in three directions based on the differences between the respiration data and the tracking data.

### C.4 Loading effect

To determine the effect of the load on the couch tracking, the loading effect was measured using the same setup as the accuracy measurement. We calculated the rms error of the loading effect in three directions based on the difference between the respiration data with and without a volunteer for 60 s cycles and between the tracking data with and without a volunteer for a certain number of cycles involving a drastic change in amplitude, such as cough data.

## III. RESULTS

### A. Overall delay time

The recorded processing time was 10 ms due to the RS‐232C communication system, and the average delay time of the CBTS was approximately 0.25 s (Table 2). Therefore, the overall delay time of the CBTS, which was calculated as the sum of the two delay times, was approximately 0.251 s.

**Table 2:** The RMS error of the accuracy measurement for the three axes of the CBTS.

*Respiration Data*	*X* ^b^	*RMS* ^a^ *Error (mm) Y* ^c^	*Z* ^d^
Sinusoidal waveform without a volunteer	2.70±1.67	2.75±1.69	5.05±3.02
Sinusoidal waveform with a volunteer	3.04±1.70	3.14±1.70	5.50±3.20
Real patient data without a volunteer	6.12±3.52	5.50±3.06	6.91±5.02
Real patient data with a volunteer	6.14±3.54	5.75±3.39	7.54±5.65

^a^ RMS=root mean square

^b^ X=vertical direction

^c^ Y=longitudinal direction

^d^ Z=lateral direction.

### B. Accuracy

**Figure 5:**
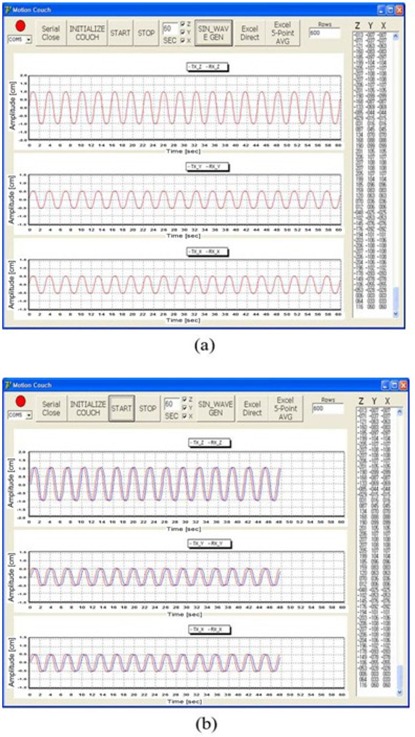
The results of the tracking data (b) according to the sinusoidal waveform (a) without a volunteer. The red and blue lines show the respiration data and the tracking data, respectively.

**Figure 6:**
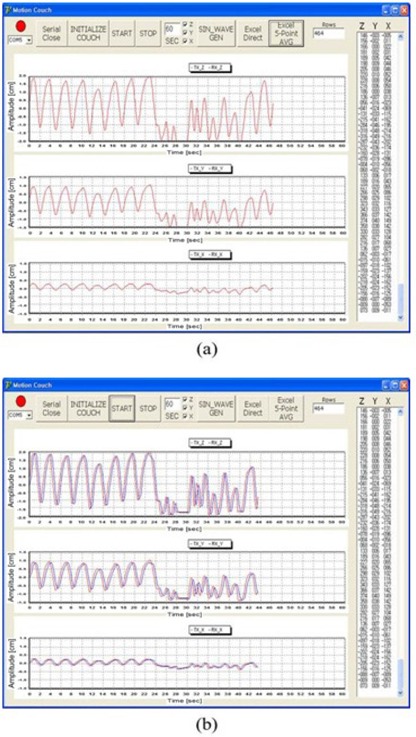
The results of the tracking data (b) according to the respiration data of real patients (a) without a volunteer. The red and blue lines show the respiration data and the tracking data, respectively.

Figures 5 and 6 show the results for the tracking data (blue line) according to the sinusoidal waveform (red line) and the various respiratory motion data without a volunteer, respectively. The tracking data agreed well with the respiration data ((Figs. 5b)and (6b). The rms error of the difference between the respiration and tracking data in three directions are summarized in Table 2. The accuracy was lowest in the Z direction with a volunteer, while the result of the rms error showed similar accuracy with and without a volunteer in the X and Y directions for a sinusoidal waveform. For real patient data, the accuracy is lowest in the Z direction with a volunteer, while the accuracy is highest in the Y direction without a volunteer.

### C. Reproducibility

As we expected, the differences between the respiration data and the tracking data were not significant. Table 3 summarizes the results of the rms error for all of the reproducibility tests. For a sinusoidal waveform, the reproducibility was lowest in the Z direction with a volunteer, while the results in the X and Y directions showed similar reproducibility with and without a volunteer. The reproducibility was lowest in the Z direction with a volunteer, while the reproducibility was highest in the X direction with a volunteer.

**Table 3:** The RMS error of the reproducibility measurement for the three axes of the CBTS.

*Respiration Data*	*X* ^b^	*RMS* ^a^ *Error (mm)*	*Y* ^c^	*Z* ^d^
Sinusoidal waveform without a volunteer	0.32±0.31	0.33±0.32	0.49±0.47
Sinusoidal waveform with a volunteer	1.01±0.85	1.03±0.87	2.12±1.79
Real patient data without a volunteer	1.44±1.43	1.38±1.35	1.96±1.93
Real patient data with a volunteer	1.87±1.83	1.20±1.19	2.34±2.30

^a^ RMS=root mean square

^b^ X=vertical direction

^c^ Y=longitudinal direction

^d^ Z=lateral direction.

### D. Loading effect

As expected, the accuracy and reproducibility were slightly reduced when the test was performed with a volunteer. Tables 2and 3 summarize the results of the rms error for all sagging tests. The loading effects were within 0.65 mm in the case of accuracy in the sinusoidal waveform (Table 2), while the result in the X direction is similar to that in the Y direction. The result in the Z direction showed the greatest effect of a volunteer when the reproducibility in the sinusoidal waveform was analyzed (Table 3). Regarding the accuracy in real patient data, the results were within 1.3 mm in all three directions (Table 2). When the reproducibility of the real patient data was measured, the results were within 1.0 mm in all directions (Table 3).

## IV. DISCUSSION

We evaluated the mechanical accuracy of the CBTS developed to compensate for tumor motion during radiation therapy. The delay time of our system was greater than that of gated beam‐delivery. Another study reported a delay of 90 ms between the recognition of a fiducial marker in a fluoroscopic image and the onset of irradiation in gated beam‐delivery.^(23)^ The delay time of our CBTS was less than the MLC‐based tracking system latency of 220 ms in the absence of prediction^(20)^ and less than the 1030 ms overall system latency for 1 Hz imaging and 570 ms for 5 Hz imaging.^(21)^ Our system needs to be further refined to reduce the delay time of our CBTS for real‐time tumor tracking radiation therapy.

The CBTS does not require staff efforts to train patient in techniques such as breath‐holding methods to obtain reproducible respiratory cycles. The CBTS can reduce treatment time compared with the beam on/off method and does not have the constraints of gating methods in which the radiation beams are delivered only within a predefined window or gate. As mentioned previously, the CBTS is a closed‐loop system that uses a PID algorithm, making it safer than other gating and MLC‐based tracking methods because when the input data exceed the predefined physical limitation — namely, the tracking velocity and the range of couch motion — the CBTS automatically stops.

A number of problems remain. The respiration data in this study were based on body surface information. However, the external breathing movement may not correspond with the true tumor or internal organ motion. Thus, the development of a CBTS that can track the tumor or internal organ motion requires further research. Further studies should be performed to analyze the correlation between the tumor motion and the respiration signal. Studies have addressed the prediction of tumor position using various algorithms and adaptive filters, such as the sequential forward floating search (SFFS) algorithm,^(24)^ adaptive neural networks,^(25)^ Kalman filters,^(26)^ the expandable ‘piston’ respiratory (EPR) model,^(27)^ the finite state automation (FSA) model,^(28)^ and the multidimensional adaptive filter (MISO predictive model).^(29)^ A model involving a respiratory motion prediction algorithm for real‐time tracking would be useful. The effect of the delay time must be further evaluated from the viewpoint of dosimetric impact and plan degradation. It has been reported that the delay time between the motion or the couch and the motion of the patient may not be significant in terms of dosimetric error for results of a phantom study.^(30)^ We are currently planning a detailed study to investigate the impact between the delay time and the plan degradation of the CBTS. Finally, more studies are required to produce a real‐time tracking radiotherapy system that can reduce the PTV margin by compensating for tumor motion due to patient respiration during radiotherapy.

## V. CONCLUSIONS

We evaluated the mechanical accuracy of the CBTS, which may be useful in clinical applications for tumor tracking in radiation therapy. The effect of the delay time must be further evaluated from the viewpoint of dosimetric impact and plan degradation. Further studies are necessary to reduce the delay time and to investigate the novel tumor motion prediction algorithm for real‐time tracking radiotherapy.

## ACKNOWLEDGMENTS

This work was supported by a Korea University Grant and by Technology Innovation Program, 10040362, Development of an Integrated Management Solution for Radiation Therapy funded by the Ministry of Knowledge Economy (MKE, Korea).
